# Outcomes of Interorganizational Networks in Canada for Chronic Disease Prevention: Insights From a Concept Mapping Study, 2015

**DOI:** 10.5888/pcd12.150297

**Published:** 2015-11-19

**Authors:** Cameron Willis, Alison Kernoghan, Barbara Riley, Janice Popp, Allan Best, H. Brinton Milward

**Affiliations:** Author Affiliations: Alison Kernoghan, Barbara Riley, Propel Centre for Population Health Impact and the School of Public Health and Health Systems, Faculty of Applied Health Sciences, University of Waterloo, Waterloo, Ontario; Janice Popp, Faculty of Social Work, University of Calgary, Calgary, Alberta; Allan Best, Centre for Clinical Epidemiology and Evaluation, Vancouver Coastal Health Research Institute, and the School of Population and Public Health, University of British Columbia, Vancouver, British Columbia; H. Brinton Milward, School of Government and Public Policy, University of Arizona, Tucson, Arizona.

## Abstract

**Introduction:**

We conducted a mixed methods study from June 2014 to March 2015 to assess the perspectives of stakeholders in networks that adopt a population approach for chronic disease prevention (CDP). The purpose of the study was to identify important and feasible outcome measures for monitoring network performance.

**Methods:**

Participants from CDP networks in Canada completed an online concept mapping exercise, which was followed by interviews with network stakeholders to further understand the findings.

**Results:**

Nine concepts were considered important outcomes of CDP networks: enhanced learning, improved use of resources, enhanced or increased relationships, improved collaborative action, network cohesion, improved system outcomes, improved population health outcomes, improved practice and policy planning, and improved intersectoral engagement. Three themes emerged from participant interviews related to measurement of the identified concepts: the methodological difficulties in measuring network outcomes, the dynamic nature of network evolution and function and implications for outcome assessment, and the challenge of measuring multisectoral engagement in CDP networks.

**Conclusion:**

Results from this study provide initial insights into concepts that can be used to describe the outcomes of networks for CDP and may offer foundations for strengthening network outcome-monitoring strategies and methodologies.

## Introduction

Interorganizational networks are groups of 3 or more organizations acting together in the pursuit of a shared vision or goal (1). Such networks are important elements of many public health efforts, including population-based chronic disease prevention (CDP) initiatives (2–5). In Canada, government and nongovernment organizations support multiple networks that aim to leverage the expertise, resources, and reach of various agents into lasting population health improvements. However, despite enthusiasm for interorganizational networks, uncertainty remains about “whether and under what conditions networks are actually performing at a level that justifies the costs of collaboration” (6). Although numerous network evaluation frameworks exist (7–10), much empirical work has focused on network structures and processes, with comparatively little research on network outcomes (1–3,11,12). The network outcomes field may be in urgent need of “reliable and valid measures of success at a network level” (9).

A focus on population-based prevention brings a unique set of contexts and challenges to the evaluation of network outcomes, such as developing realistic expectations for outcomes given the dispersed and often delayed effects of prevention activities, the difficult-to-define recipients of population health prevention efforts, and the tendency to attribute outcomes to specific organizations (3). Given these contextual factors, this exploratory project aimed to propose potentially important and feasible outcome concepts that may be useful for further enquiry as outcome monitoring strategies are developed for CDP networks.

## Methods

This study, conducted from June 2014 to March 2015, involved 2 phases: 1) concept mapping to identify outcome concepts relevant to CDP networks, and 2) key informant interviews to understand findings in light of contemporary network practice. This study received ethics clearance through a University of Waterloo Research Ethics Committee.

Concept mapping integrates qualitative processes (brainstorming and sorting) with quantitative multivariate statistical analysis, allowing groups to visually represent their ideas (13). We designed a study that used 4 steps:

**Step 1. Participant recruitment.** Thirty-three networks were nominated at a planning meeting (14) by attendees with expertise and involvement in interorganizational CDP networks. Eighteen of the 33 networks met the following inclusion criteria: they operated in Canada; they involved participants who had research, policy, and practice perspectives; and they focused on CDP population health interventions. The remaining 15 who did not meet these criteria were excluded. Each of the 18 networks included focused on 1 of 4 areas: tobacco control, physical activity, healthy eating and nutrition, or community well-being. The networks also focused on pan-Canadian, provincial, or community-based populations and were funded through a combination of support from the federal government (n = 3), provincial governments (n = 14), and nongovernment organizations (n = 2). Across the networks, sectoral involvement varied widely; each participating network involved different combinations of local public health units; community-based organizations; health care providers; government health, transport, urban planning, and education departments; charitable sectors; academic and research organizations; and individual community members. More than half of individual participants were involved in a combination of research, policy, and practice. The age of participating networks ranged from 4 to 16 years. All members of each participating network received an invitation to participate in Step 2. 

**Step 2. Online brainstorming**. Thirty-two network members completed the brainstorming exercise; 16 reported being a member of their network for more than 2 years (Table 1). By using the Concept Systems Global Max Web-based application (www.conceptsystemsglobal.com) network members provided an unlimited number of responses to the focus prompt, “A meaningful measure of network outcomes is . . .” Data were collected from July 15, 2014, through August 26, 2014. Two authors (C.W. and A.K.) removed duplicate or similar statements, separated statements into single ideas, and clarified language, resulting in a final list of 82 statements that were included in Step 3. During the statement refinement process, only a few statements were removed to retain the breadth and scope of participants’ contributions. Differences of opinion between the authors were resolved by discussion.

**Table 1:** Characteristics of Participants in Brainstorming and Sorting/Rating Groups, Concept Mapping Study, Canada, March 2015

Demographic Factor	Brainstorming Group (n = 32), n	Sorting/Rating Group (n = 11), n
**Length of network membership**		
<6 months	5	1
6–12 months	2	0
13–24 months	6	2
>24 months	16	8
No response	3	0
**Professional perspective**		
Research	3	1
Policy	0	0
Practice	7	0
Combination	20	6
Other	1	3
No response	1	1
**Organization type**		
Health care provider	16	2
Government	7	3
Nongovernment organization	4	4
Research	1	2
Education	2	0
Other	1	0
No response	1	0

**Step 3. Online sorting and rating**. Leaders from 11 networks, who were responsible for managing or coordinating network activity and who were not involved in the brainstorming activity completed the sorting and rating task. They sorted the 82 statements into discrete groupings based on similar concepts, rated the importance and the feasibility of each statement on a 5-point scale (1 = least important or least feasible; 5 = most important or most feasible), and assigned a name to each cluster. The authors discussed and reached consensus on the final cluster names.

**Step 4. Analysis.** Standard concept mapping procedures were used to analyze the relationships among statements and to generate maps (13). Statements were plotted as separate points and clustered into groups on the basis of the frequency with which they were sorted together (further methodological details are available elsewhere [13]). A stress value, which reflects the goodness-of-fit for the collected data (measures between 0.205 and 0.365 were considered acceptable), was also calculated (13). We then undertook an iterative process to identify a configuration with an interpretable set of clusters that also preserved the most detail (13). The team examined successively lower cluster solutions beginning at 13 to determine when a merger of clusters was no longer substantively reasonable. The relationship between ratings of importance and feasibility was explored through pattern-matching and calculation of a correlation coefficient (13).

Interviews with key stakeholders from CDP networks explored the consistency of concept mapping results with stakeholder experiences and implications of study findings. Ten key informants from research (n = 1), policy (n = 1), and combined settings (n = 8) were interviewed; length of employment in their current position ranged from 2 to 12 years (mean, 6 years). Interviewees were not involved in the brainstorming or sorting and rating activities. Qualitative data were analyzed using NVivo 10 (QSR International Pty Ltd) with open coding followed by inductive refinement of themes (15). Illustrative quotes from interviews were selected.

## Results

### Concept mapping

#### Individual clusters and regions

**Figure 1:**
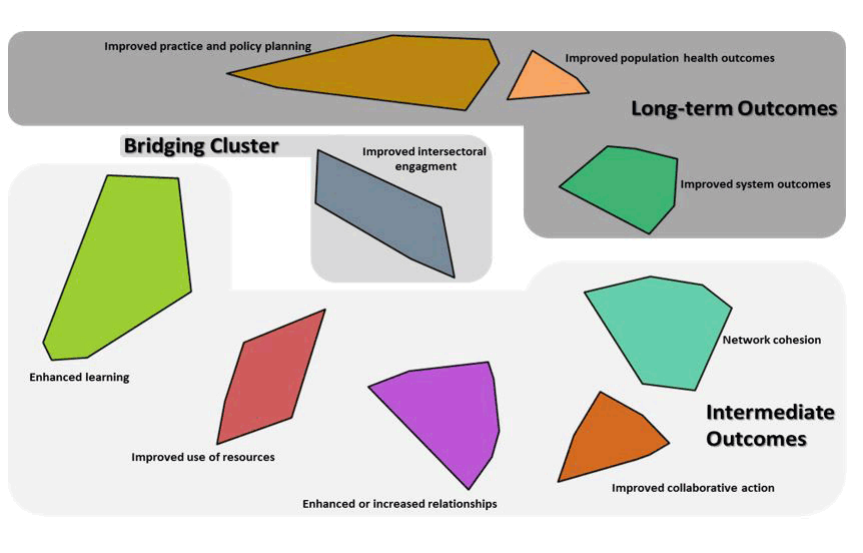
Cluster map showing regions (intermediate, bridging, and long-term outcomes).

A 9-cluster solution was determined to be the most suitable fit for this study’s data (stress value 0.280) (Figure 1). The 9 clusters were enhanced learning, improved use of resources, enhanced or increased relationships, improved collaborative action, network cohesion, improved system outcomes, improved population health outcomes, improved practice and policy planning, and improved intersectoral engagement (Table 2).Individual clusters were grouped into 3 regions: intermediate outcomes, bridging cluster, and long-term outcomes (Figure 1). The 5 clusters along the bottom of the map were considered intermediate outcomes of network performance. Population-level impacts, representing longer-term outcomes, were captured by the 3 clusters in the upper portion. The central cluster, “improved intersectoral engagement,” contains statements that highlight the importance of the coordinated involvement of multiple sectors in CDP networks. This cluster is centrally located because the statements it contains were frequently sorted with statements from all other clusters.

**Table 2:** Clusters, Number of Statements, Cluster Descriptions, and Ratings of Importance and Feasibility, Concept Mapping Study, Canada, March 2015

Cluster Name	No. of Statements	Cluster Description	Importance Rating^a^	Feasibility Rating^b^
Enhanced learning	10	Statements about increased knowledge and understanding among network members that result from network participation, both in terms of knowledge related to specific issues (eg, access to nutritious food sources) and the activities and expertise of other network member organizations. Statements in this cluster also refer to the capacity of a network to easily share knowledge among member organizations and the importance of providing a continuous flow of knowledge, facilitated by both formal and informal interorganizational connections.	4.02	3.83
Improved use of resources	5	Statements about sharing resources to reduce duplication of effort relative to both financial and nonfinancial resources. Statements in this cluster highlight the importance of network members being willing and able to describe the resources they have available for contributing to collective activities.	4.20	3.98
Enhanced or increased relationships	11	Statements related to new and improved connections that occur at the individual and organization levels as a result of network participation. Statements in this cluster refer to the ability of a network to foster complementary and reinforcing coalitions built on clear, shared objectives. This is thought to help create an environment that promotes a sense of belonging where diverse member contributions are valued.	4.02	3.70
Improved collaborative action	12	Statements focused on action that results from participation in interorganizational networks. Specific markers of meaningful collaborative action are statements of common interests and shared goals and the implementation of collaborative structures such as communities of practice.	4.05	3.56
Network cohesion	11	Statements related to concepts of trust and good will among network members and increased reciprocity among network members from engagement in network activities. Measures of network cohesion may also include clear processes for collaboratively setting priorities, the ability to engage previously disassociated organizations, and an increased capacity and confidence of organization members to recognize and work with diverse perspectives and opinions.	4.07	3.56
Improved system outcomes	9	A range of statements that relate to broad, long-term outcomes that impact the organization and delivery of population health practices. Important indicators of network outcomes in this cluster include the network’s ability to promote system-wide commitment to healthy living, such as through a common charter of healthy living. Integration was also seen as being an important system outcome influenced by network activities, including linkages among network member organizations and community-based agencies.	3.66	3.52
Improved population health outcomes	11	Statements about better health outcomes and a healthier population. This cluster also includes outcomes from networks that relate to changes in the culture and norms of populations, changes in the decision-making processes of communities, and improved alignment between prevention programs and population health needs.	4.01	3.58
Improved practice and policy planning	8	Statements that refer to outcomes such as streamlined programs and services, improved health and social policy planning, and documented changes in practice through collaboratively developed indicators of success. Such outcomes may relate to generic network functions or be linked to specific issues addressed by networks (such as food systems and environmental protection).	3.64	3.53
Improved intersectoral engagement	5	Statements that highlight the importance of the coordinated commitment and participation of multiple sectors in networks to maximize impact. Through flexible models of engagement across organizations and successful planning, their collective actions may result in change that is more effective, efficient, and sustainable.	3.64	3.35

a Rated on a 5-point scale where 1 is least important and 5 is most important.

b Rated on a 5-point scale where 1 is least feasible and 5 is most feasible.

#### Importance and feasibility ratings

**Figure 2:**
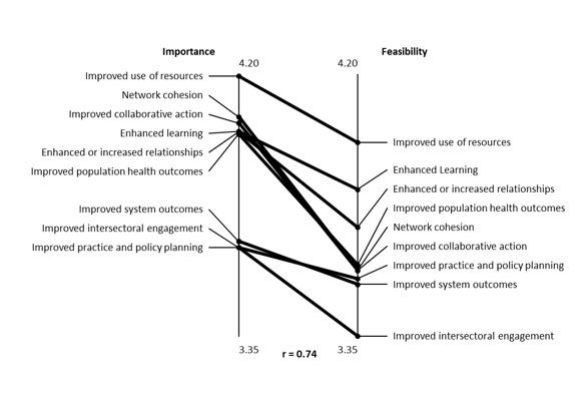
Pattern match of importance (left axis) and feasibility (right axis) demonstrating the relationship in average ratings for both characteristics across clusters.

Table 2 and Figure 2 summarize the average importance rating and feasibility rating for each cluster and describe the correlation between these ratings. Participants rated improved use of resources as the most important and most feasible outcome concept to measure and improved intersectoral engagement as the least important and least feasible measure of network outcomes. Figure 2 also demonstrates a moderately strong relationship between importance and feasibility ratings across clusters, with an overall high correlational value (*r* = 0.74).

### Stakeholder interviews

#### Concept maps and ratings

All interviewees agreed that the concept maps were comprehensive and that all outcome clusters and regions were relevant to their experience of networks. Two participants did not consider the clusters Network Cohesion and Enhanced or Increased Relationships as network outcomes. For example, network cohesion “wasn’t something we were striving for; it wasn’t an intended outcome, but a by-product of it*.*” Half of participants considered the region of long-term outcomes as the ultimate outcomes of CDP networks, and some suggested the intermediate clusters were steps in the process of achieving long-term impact.

Half of those interviewed expressed strong agreement with the importance ratings. Others questioned the relatively low importance ratings for the long-term outcome region and improved intersectoral engagement cluster, suggesting that these elements were critical for “. . . making sure that what we’re doing has an impact on the groups we’re working with.” Although half of the interviewees agreed with the feasibility ratings, opinions among the remaining 5 interviewees were highly variable, with inconsistent suggestions for higher and lower ratings for both the long-term outcomes and the improved intersectoral engagement cluster.

#### Key themes

Three themes emerged from analyses of stakeholder interviews: 1) the methodological challenges in measuring network outcomes, 2) the dynamic nature of network evolution and function and implications for outcome assessment, and 3) the importance of measures to describe intersectoral engagement in the context of CDP networks.

**Theme 1: Methodological challenges in measuring and attributing network outcomes. **Multiple interviewees referred to the difficulties in measuring network outcomes caused by multiple contributing influences (ie, political factors, available funding, government priorities). Although it can be challenging to attribute network activity to outcomes at any point along the development of a network, it may be particularly difficult when assessing long-term outcomes. As noted by one stakeholder, “longer-term outcomes are harder. That’s a practical reason why we focus on the intermediate outcomes. Those are outcomes that are within our control. . . . I can work towards improved practice and planning and overall improved system outcomes but there are other decision makers involved there.” Yet some suggested that the population impact of networks was not only important to capture but also feasible. As one research-intensive interviewee noted, “When planning is done properly at the outset of an activity of the group, the long-term outcomes should be measureable*.*” 

Interviewees also highlighted the difficulties in meeting the needs of multiple stakeholders involved in CDP networks, which by design are often multilevel and multidisciplinary. As a result, a range of individuals, organizations, and sectors operate within and around networks (eg, network leaders, members, funders, recipients), each with his or her own assumptions, expectations, and requirements of what constitutes an important and feasible outcome measure. As noted by one stakeholder, “you work in . . . an environment where organizations have different visions, different mandates, so it’s not always easy to get to common ground.” As such, the emphasis may be less on finding common ground than it is on developing a mutual understanding of needs and perspectives as they relate to network outcomes.

**Theme 2: The dynamic nature of network evolution and function and implications for outcome assessment. **Multiple interviewees suggested the identified clusters of network outcomes may take on different significance and definitions as networks evolve. Along the pathway toward high-performing CDP networks, “Intermediate outcomes are foundational in order to foster and nurture an environment that provides for upward movement long term*.*” Although networks in the early stages of development may interpret improved use of resources as relating primarily to reducing duplication, more mature networks may consider this cluster to encompass how network members are using resources for “working together more cohesively along a spectrum.” Similarly, in newly forming networks where “no one wants to give up their turf,” initial resistance to more integrated ways of working may change over time as networks foster trust and reciprocity among members.

These findings reinforce the need to link outcome measures to the stage of network development and to invest in outcome measures that realistically reflect the functions of CDP networks. For example, for CDP networks it may be particularly difficult to directly and immediately “. . . link our work to population health outcomes.” Therefore, alternative markers of network function become important, such as a network’s capacity to improve the practice and policy-planning activities of partnering organizations. Although some consider such outcomes to be long-term markers of performance, more mature networks may consider this an intermediate performance marker that helps to create the conditions for achieving population health impact. Therefore, flexibility may be required when interpreting and applying the outcome concepts identified in this study.

**Theme 3: The role of intersectoral engagement for CDP networks. **Intersectoral engagement is the coordinated participation and commitment of organizations from multiple sectors and is considered by many stakeholders to be “the foundation for everything to work” in interorganizational CDP networks. Interviewees noted the central position of intersectoral engagement with “all those other outcomes feed[ing] into it.” One example is the link between intersectoral engagement and improved use of resources. This link combines the natural focus of CDP networks on multilevel, multi-organizational participation with the utility networks offer in attracting and reaching new partners and their resources, particularly as organizations are encouraged to *“*do more with less.”

As a bridging or linking concept, measuring intersectoral engagement was considered an important step in evaluating a network’s ability to attract and retain “a multitude of sectorial players that would bring a great deal of leverage,” whose collective efforts are critical to a network’s “overall effectiveness long term.” Despite its potential importance, challenges remain in how best to capture and report intersectoral engagement. As noted by interviewees, the most useful picture of intersectoral engagement may arise from a combination of concepts, consisting of improved resource use, enhanced relationships, and increased network cohesion.

## Discussion

This study aimed to provide preliminary insights into the importance and feasibility of outcomes for measuring the performance of population-based CDP networks. Findings from this study both reinforce and extend existing knowledge of these network outcomes and their measurement. Results from this work are relevant to the design and implementation of prevention-oriented network evaluation strategies and help to surface potential domains for measuring short-term, medium-term, and long-term network outcomes for various audiences (eg, network participants, network managers, network funders). Application of this study’s findings to practical network evaluation will require tailoring through group-based item-refinement procedures.

Consistent with existing literature (7–9,16-19), this analysis identified the importance of networks for improving resource use, strengthening interorganizational relationships and cohesion, and enhancing population health status. Although existing frameworks do not explicitly include these concepts, each correlates with previously described outcome domains: network growth, reduced duplication of activities, greater integration across agencies, improved access to available resources, reduced conflict among partners, greater organizational commitment among partners, and reductions in the incidence of a particular problem. The concepts of enhanced learning and intersectoral engagement identified in our analysis are also consistent with findings from other studies (10,20). Additionally, current results highlight the temporal nature of network evolution and the need to match outcomes to the stage of network development, a finding that is consistent with existing literature related to network development and evolution (21,22), collective impact (23), and system change theories (24).

This study also offers new insights into which types of outcomes may be most important to measure for CDP networks that have a population health focus. For example, participants identified a network’s ability to influence policy and planning and contribute to the outcomes of systems promoting CDP activities as important measures of network outcomes. Previous studies of network performance frameworks did not explicitly identify these types of outcomes. This difference may relate to the composition and intended functions of CDP networks, which, unlike many service delivery networks (25,26), focus on contributing to and supporting coordinated research, policy, and practice as a means to improving population health outcomes (27,28). In these settings, more upstream measures of impact, such as improved policy and planning actions, may be expected.

There are also numerous elements included in existing frameworks that were not identified by our study, including outcomes at the community, network, and organization levels (7–10,29). The limited specificity of our results to particular outcome levels may be due to the study design, which did not attempt to identify or classify outcomes based on their level of relevance or use. An alternative focus prompt referring to community, network, or organization levels likely would have increased the specificity of statements and clusters to particular outcome levels. Future work may explore how the identified clusters can be adapted to particular levels of interest.

Variation in ratings of importance and feasibility between concept mapping participants and interviewees suggests that insights may vary depending on network roles and responsibilities. For example, concept mapping participants rated long-term network outcomes and intersectoral engagement as low on importance and feasibility, whereas interviewees rated both concepts highly. The concept mapping brainstorming participants were largely direct network members, whereas key informant interviewees also included people with broader management, policy, and research orientations for whom long-term outcomes and intersectoral collaboration may have been particularly important. Future studies that involve more participants may yield insights into how these different perspectives influence ratings of importance and feasibility.

The online concept mapping approach used in this study engaged network members from diverse geographical areas. Moreover, key informant interviews enabled in-depth exploration of concept mapping results, including preliminary validation of outcome concepts. This methodology is potentially well-suited to broader application, such as across international jurisdictions where the work of multinational networks may be particularly salient.

A limitation of this study was the relatively small number of participants. A larger group of participants drawn from different networks and settings may have identified alternative concepts, ratings, and insights. A larger sample size would also have permitted stratification of results by a range of variables, such as the perspectives of network funders, leaders, and members. Also, given the exploratory nature of this work, the qualitative analysis did not attempt to reach saturation on any particular domain, favoring capture of the key themes emergent in the data that directly linked to concept mapping results.

The concepts identified in this study provide an incremental step toward better outcome measurement for CDP networks with a population health focus. These outcomes represent potentially relevant areas in which specific indicators, tools, or techniques can be developed for describing network outcomes as CDP networks evolve. Such methodological advancements will serve as important complements to ongoing efforts to develop longitudinal case studies for tracking network evolution and change over time. As these methods continue to be developed, findings from the present study may be of use to those involved in network evaluation and management who are seeking practical guidance on ways to understand the outcomes and impact of their networks.
